# Amino­[(1*H*-benzimidazol-2-yl)amino]­methaniminium 4-methyl­benzene­sulfonate

**DOI:** 10.1107/S1600536813024975

**Published:** 2013-09-18

**Authors:** Shaaban K. Mohamed, Mehmet Akkurt, Mahmoud A. A. Elremaily, Ali. M. Ali, Mustafa R. Albayati

**Affiliations:** a Chemistry and Environmental Division, Manchester Metropolitan University, Manchester M1 5GD, England; b Chemistry Department, Faculty of Science, Minia University, 61519 El-Minia, Egypt; c Department of Physics, Faculty of Sciences, Erciyes University, 38039 Kayseri, Turkey; d Department of Organic Chemistry, Faculty of Science, Institute of Biotechnology, Granada University, Granada E-18071, Spain; e Department of Chemistry, Sohag University, 82524 Sohag, Egypt; f Kirkuk University, College of Science, Department of Chemistry, Kirkuk, Iraq

## Abstract

The asymmetric unit of the title salt, C_8_H_10_N_5_^+^·C_7_H_7_O_3_S^−^, consists of two amino­[(1*H*-benzimidazol-2-yl)amino]­meth­an­im­inium cations and two 4-methyl­benzene­sulfonate anions. The cations are each stabilized by intra­molecular N—H⋯N hydrogen bonds between the free amino groups and the imine N atoms of the benzimidazole units, forming *S*(6) ring motifs. In the crystal, cations and anions are linked by N—H⋯O and C—H⋯O hydrogen bonds, forming a three-dimensional supra­molecular framework. Two strong π–π stacking inter­actions [centroid–centroid distances = 3.4112 (14) and 3.4104 (14) Å] also occur between the centroids of the imidazole rings of like cations.

## Related literature

For the synthesis of guanidine-containing compounds, see: Wu *et al.* (2002 ▶); Hopkins *et al.* (2002 ▶); Kilburn *et al.* (2002 ▶); Manimala & Anslyn (2002 ▶). For pharmaceutical and chemical applications of guanidines, see: Han *et al.* (2008 ▶); Hannon & Anslyn (1993 ▶); Ekelund *et al.* (2001 ▶); Kovacevic & Maksic (2001 ▶); Costa *et al.* (1998 ▶). For graph-set motifs, see: Bernstein *et al.* (1995 ▶) and for standard bond lengths, see: Allen *et al.* (1987 ▶).
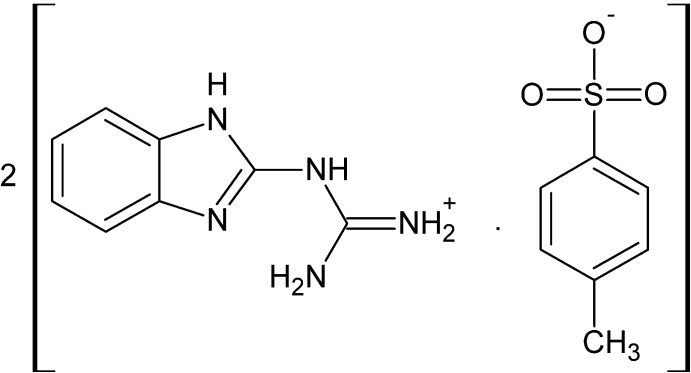


## Experimental

### 

#### Crystal data

C_8_H_10_N_5_^+^·C_7_H_7_O_3_S^−^
*M* *_r_* = 347.41
Monoclinic, 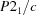

*a* = 12.3821 (4) Å
*b* = 17.8077 (7) Å
*c* = 14.5112 (5) Å
β = 90.013 (2)°
*V* = 3199.7 (2) Å^3^
*Z* = 8
Mo *K*α radiation
μ = 0.23 mm^−1^
*T* = 100 K
0.35 × 0.10 × 0.04 mm

#### Data collection

Bruker APEXII CCD diffractometer
20618 measured reflections
5679 independent reflections
4108 reflections with *I* > 2σ(*I*)
*R*_int_ = 0.044

#### Refinement

*R*[*F* ^2^ > 2σ(*F* ^2^)] = 0.044
*wR*(*F* ^2^) = 0.109
*S* = 1.05
5679 reflections
469 parameters
15 restraints
H atoms treated by a mixture of independent and constrained refinement
Δρ_max_ = 0.33 e Å^−3^
Δρ_min_ = −0.38 e Å^−3^

### 

Data collection: *APEX2* (Bruker, 2013 ▶); cell refinement: *SAINT* (Bruker, 2013 ▶); data reduction: *SAINT*; program(s) used to solve structure: *SHELXS97* (Sheldrick, 2008 ▶); program(s) used to refine structure: *SHELXL97* (Sheldrick, 2008 ▶); molecular graphics: *ORTEP-3 for Windows* (Farrugia, 2012 ▶); software used to prepare material for publication: *WinGX* (Farrugia, 2012 ▶) and *PLATON* (Spek, 2009 ▶).

## Supplementary Material

Crystal structure: contains datablock(s) global, I. DOI: 10.1107/S1600536813024975/sj5349sup1.cif

Structure factors: contains datablock(s) I. DOI: 10.1107/S1600536813024975/sj5349Isup2.hkl

Supplementary material file. DOI: 10.1107/S1600536813024975/sj5349Isup3.cml

Additional supplementary materials:  crystallographic information; 3D view; checkCIF report

## Figures and Tables

**Table 1:** Hydrogen-bond geometry (Å, °)

*D*—H⋯*A*	*D*—H	H⋯*A*	*D*⋯*A*	*D*—H⋯*A*
N1—H*N*1⋯O1^i^	0.86 (2)	2.11 (2)	2.948 (3)	166 (2)
N3—H*N*3⋯O6^ii^	0.86 (2)	1.95 (2)	2.805 (3)	173 (2)
N6—H*N*6⋯O6^iii^	0.88 (2)	2.09 (2)	2.944 (3)	164 (2)
N8—H*N*8⋯O1^iv^	0.87 (2)	1.93 (2)	2.799 (2)	177 (2)
N4—H4*A*⋯N2	0.87 (2)	1.97 (2)	2.686 (3)	139 (2)
N4—H4*B*⋯O3	0.87 (2)	2.06 (2)	2.909 (3)	166 (2)
N5—H5*A*⋯O5^ii^	0.89 (2)	1.98 (2)	2.871 (3)	176 (3)
N5—H5*B*⋯O2	0.88 (2)	1.99 (2)	2.863 (3)	169 (3)
N9—H9*A*⋯N7	0.86 (2)	1.98 (2)	2.683 (3)	138 (2)
N9—H9*B*⋯O5^v^	0.85 (2)	2.06 (2)	2.906 (3)	174 (2)
N10—H10*A*⋯O4^v^	0.88 (2)	2.00 (2)	2.863 (3)	167 (3)
N10—H10*B*⋯O3^iv^	0.89 (2)	1.99 (2)	2.872 (3)	179 (3)
C7—H7*C*⋯O4^vi^	0.98	2.53	3.283 (3)	133

Symmetry codes: (i) 
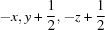
; (ii) 
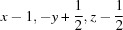
; (iii) 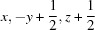; (iv) 
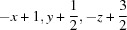
; (v) 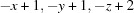; (vi) 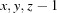.

**Fig. 1.**
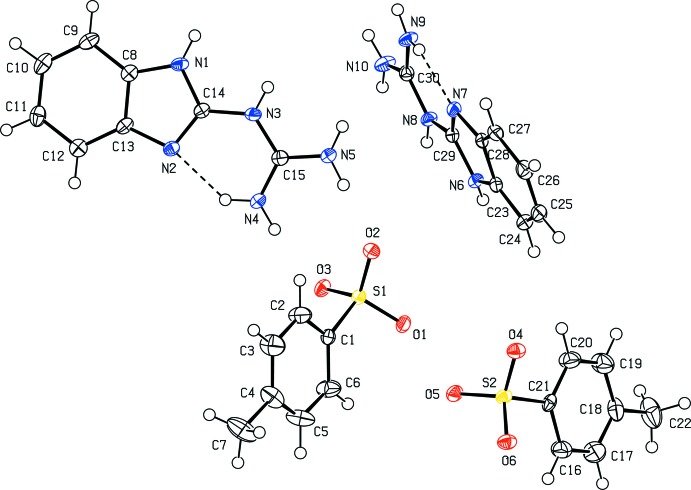
The asymmetric unit of the title compound with the atom numbering scheme. Displacement ellipsoids for non-H atoms are drawn at the 50% probability level.

**Fig. 2.**
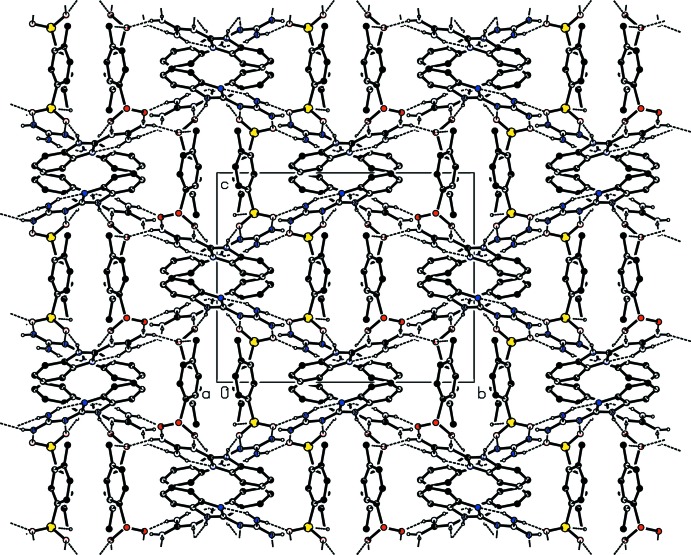
View of the packing and hydrogen bonding (dashed lines) along the a axis of the title compound.

## supplementary crystallographic information

### 1. Comment

Guanidines are structurally novel molecules reported to exhibit remarkable biological and pharmacological activities, which are affected by the guanidine functionality (Han *et al.*, 2008; Hannon & Anslyn, 1993). Guanidino-containing drugs such as metaiodobenzylguanidine, MIBG, and methylglyoxalbisguanylhydrazone, MGBG, were shown several decades ago to have antitumor properties and have been subjected to intense preclinical and clinical evaluation (Ekelund *et al.*, 2001). Guanidines are also known as useful basic catalysts (Kovacevic & Maksic, 2001; Costa *et al.*, 1998). The synthesis of guanidine derivatives has also attracted continued research interests in recent years, resulting in many new efficient synthetic methods and guanidinylation reagents for different classes of guanidine compounds (Wu *et al.*, 2002; Hopkins *et al.*, 2002; Kilburn *et al.*, 2002; Manimala & Anslyn, 2002). Against this background, we report herein the the synthesis and crystal structure of the title compound.

As seen as in Fig. 1, the asymmetric unit contains two amino(1*H*-benzimidazol-2-ylamino)methaniminium) cations and two 4-methylbenzenesulfonate anions. The bond lengths in the title compound are within the normal range (Allen *et al.*, 1987).

In the cations, intramolecular N4—H4A···N2 and N9—H9A···N7 hydrogen bonds generate six-membered S(6) rings in each cation (Bernstein *et al.*, 1995). In the crystal, a three-dimensional supramolecular framework is formed *via* intermolecular N—H···O and C—H···O hydrogen bonds between the cations and anions (Table 1, Fig. 2). Furthermore, two strong π-π stacking interactions [Cg1···Cg1 (-*x*, 1 - *y*, -*z*) = 3.4112 (14) Å and Cg4···Cg4 (1 - *x*, 1 - *y*, 2 - *z*) = 3.4104 (14) Å] also occur between the imidazole rings of like cations (Cg1 and Cg4 are the centroids of the N1/C8/C13/N2/C14 and N6/C23/C28/N7/C29 ring respectively).

### 2. Experimental

A mixture of 175 mg (1 mmol) 1-(1*H*-benzimidazol-2-yl)guanidine and 191 mg (1 mmol) of 4-methylbenzenesulfonyl chloride was heated under reflux in 50 ml ethanol together with few drops of triethylamine for 6 h. The solid product started to be deposited during heating and filtered off after completion. The crude solid was washed with ethanol and recrystallized to afford colourless plates suitable for X-ray difraction (*M*.p. 539–541 K).

### 3. Refinement

The C-bound H atoms were placed at geometrically idealized positions with C—H = 0.95 and 0.98 Å for aromatic and methyl H-atoms, respectively. The C-bound H-atoms were refined using a riding model with *U*_iso_(H) = 1.2*U*_eq_(C_aromatic_) and 1.5*U*_eq_(C_methyl_). The N-bound H atoms were located in a difference Fourier map and their positions were refined with distance restraints [N—H = 0.88 (2) Å] and with *U*_iso_(H) = 1.2*U*_eq_(N). The presence of pseudosymmetry in the structure suggests the orthorhombic space group Pbcn, but attempts to refine the structure in this space group resulted in an unsatisfactory model.

### Crystal data

**Table:**

C_8_H_10_N_5_^+^·C_7_H_7_O_3_S^−^	*F*(000) = 1456
*M**_r_* = 347.41	*D*_x_ = 1.442 Mg m^−^^3^
Monoclinic, *P*2_1_/*c*	Mo *K*α radiation, λ = 0.71073 Å
Hall symbol: -P 2ybc	Cell parameters from 9624 reflections
*a* = 12.3821 (4) Å	θ = 2.5–25.1°
*b* = 17.8077 (7) Å	µ = 0.23 mm^−^^1^
*c* = 14.5112 (5) Å	*T* = 100 K
β = 90.013 (2)°	Plate, colourless
*V* = 3199.7 (2) Å^3^	0.35 × 0.10 × 0.04 mm
*Z* = 8	

### Data collection

**Table:**

Bruker APEXII CCD diffractometer	4108 reflections with *I* > 2σ(*I*)
Radiation source: sealed tube	*R*_int_ = 0.044
Graphite monochromator	θ_max_ = 25.1°, θ_min_ = 2.5°
φ and ω scans	*h* = −14→14
20618 measured reflections	*k* = −17→21
5679 independent reflections	*l* = −17→17

### Refinement

**Table:**

Refinement on *F*^2^	15 restraints
Least-squares matrix: full	H atoms treated by a mixture of independent and constrained refinement
*R*[*F*^2^ > 2σ(*F*^2^)] = 0.044	W = 1/[Σ^2^(*F*O^2^) + (0.0495*P*)^2^ + 1.3622*P*] where *P* = (*F*O^2^ + 2*F*C^2^)/3
*wR*(*F*^2^) = 0.109	(Δ/σ)_max_ < 0.001
*S* = 1.05	Δρ_max_ = 0.33 e Å^−^^3^
5679 reflections	Δρ_min_ = −0.38 e Å^−^^3^
469 parameters	

### Special details

**Table:**

Geometry. Bond distances, angles *etc*. have been calculated using the rounded fractional coordinates. All su's are estimated from the variances of the (full) variance-covariance matrix. The cell e.s.d.'s are taken into account in the estimation of distances, angles and torsion angles
Refinement. Refinement on *F*^2^ for ALL reflections except those flagged by the user for potential systematic errors. Weighted *R*-factors *wR* and all goodnesses of fit *S* are based on *F*^2^, conventional *R*-factors *R* are based on *F*, with *F* set to zero for negative *F*^2^. The observed criterion of *F*^2^ > σ(*F*^2^) is used only for calculating -*R*-factor-obs *etc*. and is not relevant to the choice of reflections for refinement. *R*-factors based on *F*^2^ are statistically about twice as large as those based on *F*, and *R*-factors based on ALL data will be even larger.

### Fractional atomic coordinates and isotropic or equivalent isotropic displacement parameters (Å^2^)

**Table:**

	*x*	*y*	*z*	*U*_iso_*/*U*_eq_	
N1	−0.06286 (16)	0.53699 (11)	0.14245 (14)	0.0175 (6)	
N2	0.09357 (15)	0.48502 (11)	0.09774 (13)	0.0169 (6)	
N3	−0.03173 (16)	0.40955 (11)	0.18217 (14)	0.0180 (6)	
N4	0.12756 (17)	0.34452 (13)	0.15854 (15)	0.0213 (7)	
N5	−0.01669 (17)	0.28656 (13)	0.22877 (15)	0.0228 (7)	
C8	−0.00584 (19)	0.59345 (14)	0.09792 (15)	0.0175 (8)	
C9	−0.0290 (2)	0.66825 (15)	0.08044 (17)	0.0225 (8)	
C10	0.0487 (2)	0.70888 (15)	0.03340 (18)	0.0267 (9)	
C11	0.1448 (2)	0.67604 (14)	0.00378 (18)	0.0239 (8)	
C12	0.1676 (2)	0.60121 (14)	0.02124 (16)	0.0210 (8)	
C13	0.09108 (19)	0.56002 (14)	0.06984 (16)	0.0169 (7)	
C14	0.00195 (18)	0.47560 (13)	0.14059 (16)	0.0158 (7)	
C15	0.02761 (19)	0.34569 (14)	0.18891 (16)	0.0181 (8)	
N6	0.56311 (16)	0.46290 (12)	1.14251 (14)	0.0176 (7)	
N7	0.40627 (15)	0.51515 (11)	1.09786 (13)	0.0165 (6)	
N8	0.53226 (16)	0.59052 (11)	1.18216 (14)	0.0174 (6)	
N9	0.37236 (17)	0.65557 (12)	1.15834 (15)	0.0206 (7)	
N10	0.51667 (17)	0.71349 (13)	1.22845 (15)	0.0239 (7)	
C23	0.50569 (19)	0.40666 (14)	1.09794 (16)	0.0174 (7)	
C24	0.5292 (2)	0.33155 (14)	1.08030 (18)	0.0230 (8)	
C25	0.4513 (2)	0.29117 (15)	1.03352 (18)	0.0256 (8)	
C26	0.3551 (2)	0.32380 (14)	1.00391 (18)	0.0240 (8)	
C27	0.33247 (19)	0.39879 (14)	1.02117 (16)	0.0208 (8)	
C28	0.40902 (19)	0.43994 (14)	1.06964 (16)	0.0170 (7)	
C29	0.49835 (18)	0.52464 (14)	1.14061 (15)	0.0162 (8)	
C30	0.47239 (19)	0.65446 (14)	1.18866 (16)	0.0168 (8)	
S1	0.21220 (5)	0.14511 (3)	0.19245 (4)	0.0171 (2)	
O1	0.25453 (12)	0.08701 (10)	0.25384 (11)	0.0209 (5)	
O2	0.09531 (12)	0.14896 (9)	0.19288 (11)	0.0208 (5)	
O3	0.26233 (12)	0.21792 (9)	0.21165 (11)	0.0199 (5)	
C1	0.25080 (18)	0.12068 (13)	0.07911 (17)	0.0182 (8)	
C2	0.1867 (2)	0.14390 (16)	0.00683 (19)	0.0307 (9)	
C3	0.2188 (2)	0.13075 (18)	−0.08296 (19)	0.0363 (10)	
C4	0.3145 (2)	0.09439 (17)	−0.10240 (19)	0.0312 (9)	
C5	0.3776 (2)	0.07199 (19)	−0.0290 (2)	0.0385 (10)	
C6	0.3467 (2)	0.08403 (17)	0.06154 (19)	0.0321 (9)	
C7	0.3494 (2)	0.0806 (2)	−0.2003 (2)	0.0464 (13)	
S2	0.71207 (5)	0.14502 (3)	0.80768 (4)	0.0170 (2)	
O4	0.59550 (13)	0.14897 (10)	0.80726 (11)	0.0211 (5)	
O5	0.76233 (12)	0.21802 (9)	0.78844 (11)	0.0210 (5)	
O6	0.75459 (13)	0.08684 (10)	0.74612 (11)	0.0218 (5)	
C16	0.8464 (2)	0.08440 (17)	0.93848 (19)	0.0330 (9)	
C17	0.8775 (2)	0.07195 (19)	1.0293 (2)	0.0397 (10)	
C18	0.8143 (2)	0.09440 (17)	1.10254 (18)	0.0295 (9)	
C19	0.7187 (2)	0.13093 (18)	1.08268 (19)	0.0353 (10)	
C20	0.6868 (2)	0.14404 (16)	0.99331 (19)	0.0301 (9)	
C21	0.75051 (19)	0.12081 (14)	0.92102 (16)	0.0178 (8)	
C22	0.8491 (2)	0.0808 (2)	1.2004 (2)	0.0486 (13)	
HN1	−0.1196 (16)	0.5434 (14)	0.1755 (16)	0.0210*	
HN3	−0.0980 (14)	0.4078 (15)	0.1986 (17)	0.0220*	
H4A	0.148 (2)	0.3840 (12)	0.1274 (17)	0.0260*	
H4B	0.1687 (18)	0.3051 (12)	0.1641 (18)	0.0260*	
H5A	−0.0860 (14)	0.2874 (16)	0.2457 (18)	0.0270*	
H5B	0.021 (2)	0.2448 (12)	0.2250 (19)	0.0270*	
H9	−0.09500	0.69050	0.09980	0.0270*	
H10	0.03620	0.76050	0.02100	0.0320*	
H11	0.19580	0.70560	−0.02910	0.0290*	
H12	0.23300	0.57890	0.00080	0.0250*	
HN6	0.6213 (15)	0.4571 (15)	1.1766 (16)	0.0210*	
HN8	0.5993 (14)	0.5904 (15)	1.2000 (16)	0.0210*	
H9A	0.353 (2)	0.6174 (12)	1.1260 (16)	0.0250*	
H9B	0.3349 (19)	0.6944 (12)	1.1706 (18)	0.0250*	
H10A	0.478 (2)	0.7546 (12)	1.2263 (19)	0.0290*	
H10B	0.5849 (14)	0.7141 (16)	1.2466 (18)	0.0290*	
H24	0.59530	0.30930	1.09940	0.0280*	
H25	0.46380	0.23950	1.02110	0.0310*	
H26	0.30410	0.29410	0.97130	0.0290*	
H27	0.26710	0.42120	1.00060	0.0250*	
H2	0.12040	0.16900	0.01870	0.0370*	
H3	0.17410	0.14710	−0.13220	0.0440*	
H5	0.44430	0.04750	−0.04100	0.0460*	
H6	0.39100	0.06730	0.11090	0.0390*	
H7A	0.33560	0.12570	−0.23730	0.0690*	
H7B	0.30860	0.03830	−0.22580	0.0690*	
H7C	0.42680	0.06900	−0.20160	0.0690*	
H16	0.89080	0.06800	0.88910	0.0400*	
H17	0.94410	0.04730	1.04120	0.0480*	
H19	0.67400	0.14740	1.13190	0.0420*	
H20	0.62060	0.16920	0.98150	0.0360*	
H22A	0.80090	0.04390	1.22900	0.0730*	
H22B	0.84580	0.12800	1.23500	0.0730*	
H22C	0.92330	0.06170	1.20100	0.0730*	

### Atomic displacement parameters (Å^2^)

**Table:**

	*U*^11^	*U*^22^	*U*^33^	*U*^12^	*U*^13^	*U*^23^
N1	0.0175 (11)	0.0148 (11)	0.0202 (11)	0.0030 (9)	0.0011 (8)	−0.0053 (9)
N2	0.0175 (11)	0.0146 (11)	0.0187 (10)	0.0009 (9)	−0.0013 (8)	−0.0022 (9)
N3	0.0129 (10)	0.0180 (12)	0.0232 (11)	0.0025 (9)	0.0019 (8)	0.0001 (9)
N4	0.0207 (12)	0.0157 (13)	0.0275 (12)	0.0043 (9)	0.0027 (9)	0.0042 (10)
N5	0.0198 (11)	0.0176 (13)	0.0311 (12)	0.0049 (10)	0.0052 (9)	0.0061 (10)
C8	0.0206 (13)	0.0171 (14)	0.0149 (12)	0.0004 (10)	−0.0044 (9)	−0.0027 (10)
C9	0.0251 (14)	0.0175 (14)	0.0249 (13)	0.0049 (11)	−0.0062 (11)	−0.0048 (11)
C10	0.0340 (16)	0.0160 (15)	0.0300 (14)	0.0006 (12)	−0.0085 (12)	0.0008 (12)
C11	0.0266 (14)	0.0202 (14)	0.0250 (13)	−0.0053 (11)	−0.0048 (11)	0.0034 (12)
C12	0.0209 (13)	0.0218 (15)	0.0204 (13)	−0.0014 (11)	−0.0038 (10)	−0.0014 (11)
C13	0.0204 (13)	0.0124 (13)	0.0178 (12)	0.0007 (10)	−0.0059 (10)	−0.0037 (10)
C14	0.0170 (12)	0.0141 (13)	0.0162 (12)	0.0004 (10)	−0.0025 (9)	−0.0028 (10)
C15	0.0180 (13)	0.0197 (15)	0.0167 (12)	0.0020 (11)	−0.0008 (10)	−0.0011 (10)
N6	0.0163 (11)	0.0157 (12)	0.0208 (11)	0.0034 (9)	−0.0005 (8)	0.0031 (9)
N7	0.0190 (11)	0.0135 (11)	0.0171 (10)	0.0001 (9)	0.0012 (8)	0.0004 (9)
N8	0.0142 (10)	0.0154 (12)	0.0227 (11)	0.0021 (9)	−0.0021 (8)	0.0005 (9)
N9	0.0200 (12)	0.0127 (12)	0.0290 (12)	0.0035 (9)	−0.0014 (9)	−0.0056 (10)
N10	0.0198 (12)	0.0177 (13)	0.0342 (13)	0.0039 (10)	−0.0042 (10)	−0.0051 (10)
C23	0.0201 (13)	0.0146 (13)	0.0174 (12)	−0.0003 (10)	0.0049 (10)	0.0027 (10)
C24	0.0250 (14)	0.0169 (14)	0.0272 (14)	0.0042 (11)	0.0062 (11)	0.0025 (11)
C25	0.0318 (15)	0.0137 (14)	0.0312 (15)	−0.0006 (12)	0.0112 (12)	−0.0005 (12)
C26	0.0268 (14)	0.0190 (14)	0.0262 (14)	−0.0059 (12)	0.0044 (11)	−0.0033 (12)
C27	0.0185 (13)	0.0205 (15)	0.0234 (13)	−0.0014 (11)	0.0050 (10)	0.0014 (11)
C28	0.0203 (13)	0.0150 (13)	0.0156 (12)	0.0009 (10)	0.0052 (10)	0.0030 (10)
C29	0.0178 (13)	0.0161 (14)	0.0148 (12)	0.0009 (10)	0.0021 (9)	0.0030 (10)
C30	0.0184 (13)	0.0166 (14)	0.0153 (12)	−0.0007 (10)	0.0007 (10)	0.0001 (10)
S1	0.0165 (3)	0.0142 (3)	0.0205 (3)	0.0006 (2)	0.0007 (2)	0.0022 (3)
O1	0.0190 (9)	0.0171 (10)	0.0267 (9)	0.0007 (7)	−0.0013 (7)	0.0067 (8)
O2	0.0166 (9)	0.0212 (10)	0.0245 (9)	0.0000 (7)	0.0024 (7)	0.0013 (8)
O3	0.0222 (9)	0.0130 (9)	0.0245 (9)	−0.0008 (7)	−0.0026 (7)	0.0001 (7)
C1	0.0182 (13)	0.0117 (13)	0.0246 (13)	−0.0023 (10)	0.0032 (10)	0.0000 (11)
C2	0.0279 (15)	0.0370 (17)	0.0272 (14)	0.0119 (13)	0.0009 (12)	−0.0022 (13)
C3	0.0344 (17)	0.049 (2)	0.0254 (15)	0.0067 (14)	−0.0032 (12)	−0.0034 (14)
C4	0.0267 (15)	0.0379 (18)	0.0291 (15)	−0.0119 (13)	0.0026 (12)	−0.0130 (13)
C5	0.0242 (15)	0.051 (2)	0.0404 (18)	0.0084 (14)	0.0052 (13)	−0.0144 (15)
C6	0.0251 (15)	0.0411 (19)	0.0302 (15)	0.0109 (13)	−0.0043 (12)	−0.0029 (14)
C7	0.0329 (17)	0.073 (3)	0.0332 (17)	−0.0122 (16)	0.0085 (13)	−0.0214 (17)
S2	0.0163 (3)	0.0140 (3)	0.0206 (3)	0.0006 (2)	−0.0008 (2)	−0.0022 (3)
O4	0.0170 (9)	0.0209 (10)	0.0253 (9)	0.0016 (7)	−0.0014 (7)	−0.0022 (8)
O5	0.0213 (9)	0.0137 (10)	0.0279 (9)	−0.0003 (7)	0.0010 (7)	−0.0009 (8)
O6	0.0196 (9)	0.0186 (10)	0.0271 (9)	0.0019 (7)	0.0010 (7)	−0.0054 (8)
C16	0.0260 (15)	0.0418 (19)	0.0311 (15)	0.0109 (13)	0.0044 (12)	0.0083 (14)
C17	0.0252 (16)	0.053 (2)	0.0410 (18)	0.0095 (14)	−0.0048 (13)	0.0168 (16)
C18	0.0275 (15)	0.0349 (18)	0.0262 (14)	−0.0088 (13)	−0.0032 (11)	0.0110 (13)
C19	0.0334 (16)	0.048 (2)	0.0245 (15)	0.0070 (14)	0.0019 (12)	0.0014 (14)
C20	0.0271 (15)	0.0367 (17)	0.0266 (14)	0.0116 (13)	−0.0002 (11)	0.0016 (13)
C21	0.0194 (13)	0.0114 (13)	0.0226 (13)	−0.0029 (10)	−0.0044 (10)	0.0016 (10)
C22	0.0365 (18)	0.075 (3)	0.0342 (17)	−0.0139 (17)	−0.0102 (14)	0.0238 (18)

### Geometric parameters (Å, º)

**Table:**

S1—O2	1.4490 (16)	C9—H9	0.9500
S1—O3	1.4642 (17)	C10—H10	0.9500
S1—O1	1.4624 (18)	C11—H11	0.9500
S1—C1	1.767 (3)	C12—H12	0.9500
S2—C21	1.766 (2)	C23—C24	1.393 (4)
S2—O6	1.4659 (18)	C23—C28	1.397 (3)
S2—O5	1.4681 (17)	C24—C25	1.381 (4)
S2—O4	1.4451 (17)	C25—C26	1.393 (4)
N1—C14	1.356 (3)	C26—C27	1.387 (4)
N1—C8	1.388 (3)	C27—C28	1.389 (3)
N2—C14	1.305 (3)	C24—H24	0.9500
N2—C13	1.396 (3)	C25—H25	0.9500
N3—C15	1.357 (3)	C26—H26	0.9500
N3—C14	1.386 (3)	C27—H27	0.9500
N4—C15	1.314 (3)	C1—C6	1.379 (3)
N5—C15	1.321 (3)	C1—C2	1.379 (4)
N1—HN1	0.86 (2)	C2—C3	1.382 (4)
N3—HN3	0.855 (18)	C3—C4	1.380 (4)
N4—H4A	0.87 (2)	C4—C7	1.505 (4)
N4—H4B	0.87 (2)	C4—C5	1.380 (4)
N5—H5A	0.893 (18)	C5—C6	1.385 (4)
N5—H5B	0.88 (2)	C2—H2	0.9500
N6—C29	1.361 (3)	C3—H3	0.9500
N6—C23	1.388 (3)	C5—H5	0.9500
N7—C28	1.401 (3)	C6—H6	0.9500
N7—C29	1.309 (3)	C7—H7B	0.9800
N8—C29	1.384 (3)	C7—H7C	0.9800
N8—C30	1.362 (3)	C7—H7A	0.9800
N9—C30	1.315 (3)	C16—C17	1.391 (4)
N10—C30	1.319 (3)	C16—C21	1.376 (4)
N6—HN6	0.88 (2)	C17—C18	1.379 (4)
N8—HN8	0.870 (18)	C18—C19	1.381 (4)
N9—H9B	0.85 (2)	C18—C22	1.504 (4)
N9—H9A	0.86 (2)	C19—C20	1.376 (4)
N10—H10B	0.885 (19)	C20—C21	1.376 (4)
N10—H10A	0.88 (2)	C16—H16	0.9500
C8—C9	1.386 (4)	C17—H17	0.9500
C8—C13	1.400 (3)	C19—H19	0.9500
C9—C10	1.384 (4)	C20—H20	0.9500
C10—C11	1.394 (4)	C22—H22A	0.9800
C11—C12	1.386 (4)	C22—H22B	0.9800
C12—C13	1.391 (3)	C22—H22C	0.9800
			
O2—S1—C1	106.62 (10)	C25—C26—C27	121.2 (2)
O3—S1—C1	106.28 (10)	C26—C27—C28	117.5 (2)
O1—S1—O3	111.02 (10)	N7—C28—C23	109.9 (2)
O1—S1—C1	107.21 (10)	N7—C28—C27	129.5 (2)
O1—S1—O2	112.88 (9)	C23—C28—C27	120.6 (2)
O2—S1—O3	112.38 (9)	N7—C29—N8	125.5 (2)
O6—S2—C21	107.36 (11)	N6—C29—N7	114.7 (2)
O4—S2—O5	112.31 (10)	N6—C29—N8	119.8 (2)
O5—S2—C21	106.21 (11)	N8—C30—N10	118.1 (2)
O5—S2—O6	110.96 (10)	N8—C30—N9	120.2 (2)
O4—S2—O6	113.00 (10)	N9—C30—N10	121.8 (2)
O4—S2—C21	106.55 (11)	C25—C24—H24	122.00
C8—N1—C14	105.87 (19)	C23—C24—H24	122.00
C13—N2—C14	104.01 (19)	C24—C25—H25	119.00
C14—N3—C15	125.4 (2)	C26—C25—H25	119.00
C8—N1—HN1	125.5 (17)	C25—C26—H26	119.00
C14—N1—HN1	127.1 (16)	C27—C26—H26	119.00
C14—N3—HN3	116.2 (18)	C28—C27—H27	121.00
C15—N3—HN3	118.0 (18)	C26—C27—H27	121.00
C15—N4—H4A	115.7 (16)	S1—C1—C6	121.45 (19)
C15—N4—H4B	122.2 (15)	S1—C1—C2	118.58 (18)
H4A—N4—H4B	122 (2)	C2—C1—C6	119.8 (2)
C15—N5—H5A	120.4 (18)	C1—C2—C3	120.1 (2)
H5A—N5—H5B	123 (2)	C2—C3—C4	121.3 (3)
C15—N5—H5B	115.2 (16)	C5—C4—C7	121.3 (2)
C23—N6—C29	105.77 (19)	C3—C4—C5	117.7 (3)
C28—N7—C29	103.93 (19)	C3—C4—C7	121.1 (2)
C29—N8—C30	125.0 (2)	C4—C5—C6	122.1 (2)
C29—N6—HN6	126.1 (17)	C1—C6—C5	119.1 (2)
C23—N6—HN6	126.6 (18)	C1—C2—H2	120.00
C29—N8—HN8	114.7 (17)	C3—C2—H2	120.00
C30—N8—HN8	120.1 (18)	C4—C3—H3	119.00
C30—N9—H9B	117.1 (16)	C2—C3—H3	119.00
C30—N9—H9A	115.7 (16)	C4—C5—H5	119.00
H9A—N9—H9B	127 (2)	C6—C5—H5	119.00
C30—N10—H10B	122.5 (18)	C1—C6—H6	120.00
H10A—N10—H10B	122 (2)	C5—C6—H6	120.00
C30—N10—H10A	115.1 (16)	H7B—C7—H7C	110.00
C9—C8—C13	122.2 (2)	C4—C7—H7A	109.00
N1—C8—C13	105.3 (2)	C4—C7—H7B	110.00
N1—C8—C9	132.5 (2)	C4—C7—H7C	109.00
C8—C9—C10	116.7 (2)	H7A—C7—H7B	109.00
C9—C10—C11	121.8 (2)	H7A—C7—H7C	109.00
C10—C11—C12	121.4 (2)	C17—C16—C21	119.2 (2)
C11—C12—C13	117.5 (2)	C16—C17—C18	121.8 (2)
N2—C13—C8	110.0 (2)	C17—C18—C19	117.5 (2)
C8—C13—C12	120.5 (2)	C17—C18—C22	121.2 (2)
N2—C13—C12	129.6 (2)	C19—C18—C22	121.2 (2)
N1—C14—N3	119.8 (2)	C18—C19—C20	121.5 (2)
N1—C14—N2	114.9 (2)	C19—C20—C21	120.2 (2)
N2—C14—N3	125.3 (2)	S2—C21—C16	121.26 (19)
N4—C15—N5	121.7 (2)	S2—C21—C20	118.83 (19)
N3—C15—N5	118.3 (2)	C16—C21—C20	119.7 (2)
N3—C15—N4	119.9 (2)	C17—C16—H16	120.00
C10—C9—H9	122.00	C21—C16—H16	120.00
C8—C9—H9	122.00	C16—C17—H17	119.00
C11—C10—H10	119.00	C18—C17—H17	119.00
C9—C10—H10	119.00	C18—C19—H19	119.00
C10—C11—H11	119.00	C20—C19—H19	119.00
C12—C11—H11	119.00	C19—C20—H20	120.00
C13—C12—H12	121.00	C21—C20—H20	120.00
C11—C12—H12	121.00	C18—C22—H22A	109.00
N6—C23—C24	132.2 (2)	C18—C22—H22B	109.00
N6—C23—C28	105.6 (2)	C18—C22—H22C	109.00
C24—C23—C28	122.2 (2)	H22A—C22—H22B	109.00
C23—C24—C25	116.4 (2)	H22A—C22—H22C	110.00
C24—C25—C26	122.1 (2)	H22B—C22—H22C	109.00
			
O1—S1—C1—C2	152.0 (2)	C13—C8—C9—C10	0.1 (4)
O2—S1—C1—C2	30.8 (2)	N1—C8—C9—C10	179.8 (2)
O3—S1—C1—C2	−89.3 (2)	N1—C8—C13—C12	179.1 (2)
O1—S1—C1—C6	−32.8 (2)	C8—C9—C10—C11	1.0 (4)
O2—S1—C1—C6	−153.9 (2)	C9—C10—C11—C12	−0.9 (4)
O3—S1—C1—C6	86.0 (2)	C10—C11—C12—C13	−0.2 (4)
O5—S2—C21—C20	89.1 (2)	C11—C12—C13—C8	1.2 (3)
O6—S2—C21—C20	−152.1 (2)	C11—C12—C13—N2	−179.0 (2)
O6—S2—C21—C16	32.8 (2)	C28—C23—C24—C25	−0.4 (4)
O4—S2—C21—C16	154.2 (2)	N6—C23—C24—C25	179.7 (2)
O5—S2—C21—C16	−85.9 (2)	C24—C23—C28—N7	179.0 (2)
O4—S2—C21—C20	−30.8 (2)	N6—C23—C28—N7	−1.0 (3)
C14—N1—C8—C9	−178.3 (3)	N6—C23—C28—C27	179.2 (2)
C14—N1—C8—C13	1.4 (2)	C24—C23—C28—C27	−0.8 (4)
C8—N1—C14—N3	178.5 (2)	C23—C24—C25—C26	1.1 (4)
C8—N1—C14—N2	−1.8 (3)	C24—C25—C26—C27	−0.8 (4)
C13—N2—C14—N3	−179.0 (2)	C25—C26—C27—C28	−0.4 (4)
C14—N2—C13—C8	−0.3 (3)	C26—C27—C28—C23	1.2 (3)
C13—N2—C14—N1	1.3 (3)	C26—C27—C28—N7	−178.6 (2)
C14—N2—C13—C12	180.0 (2)	S1—C1—C2—C3	175.1 (2)
C14—N3—C15—N5	−178.4 (2)	C2—C1—C6—C5	0.8 (4)
C15—N3—C14—N2	3.6 (4)	C6—C1—C2—C3	−0.3 (4)
C14—N3—C15—N4	3.5 (4)	S1—C1—C6—C5	−174.4 (2)
C15—N3—C14—N1	−176.6 (2)	C1—C2—C3—C4	0.1 (4)
C23—N6—C29—N8	178.4 (2)	C2—C3—C4—C7	−179.7 (3)
C23—N6—C29—N7	−1.5 (3)	C2—C3—C4—C5	−0.4 (4)
C29—N6—C23—C24	−178.6 (3)	C3—C4—C5—C6	1.0 (5)
C29—N6—C23—C28	1.5 (2)	C7—C4—C5—C6	−179.8 (3)
C28—N7—C29—N8	−179.1 (2)	C4—C5—C6—C1	−1.1 (5)
C29—N7—C28—C27	180.0 (3)	C17—C16—C21—C20	−0.3 (4)
C29—N7—C28—C23	0.2 (3)	C17—C16—C21—S2	174.8 (2)
C28—N7—C29—N6	0.8 (3)	C21—C16—C17—C18	0.7 (5)
C30—N8—C29—N6	−176.5 (2)	C16—C17—C18—C19	−0.8 (5)
C29—N8—C30—N9	3.6 (4)	C16—C17—C18—C22	−179.8 (3)
C30—N8—C29—N7	3.4 (4)	C17—C18—C19—C20	0.5 (4)
C29—N8—C30—N10	−178.4 (2)	C22—C18—C19—C20	179.5 (3)
N1—C8—C13—N2	−0.7 (3)	C18—C19—C20—C21	−0.1 (4)
C9—C8—C13—N2	179.0 (2)	C19—C20—C21—C16	0.0 (4)
C9—C8—C13—C12	−1.2 (4)	C19—C20—C21—S2	−175.2 (2)

### Hydrogen-bond geometry (Å, º)

**Table:**

*D*—H···*A*	*D*—H	H···*A*	*D*···*A*	*D*—H···*A*
N1—H*N*1···O1^i^	0.86 (2)	2.11 (2)	2.948 (3)	166 (2)
N3—H*N*3···O6^ii^	0.86 (2)	1.95 (2)	2.805 (3)	173 (2)
N6—H*N*6···O6^iii^	0.88 (2)	2.09 (2)	2.944 (3)	164 (2)
N8—H*N*8···O1^iv^	0.87 (2)	1.93 (2)	2.799 (2)	177 (2)
N4—H4*A*···N2	0.87 (2)	1.97 (2)	2.686 (3)	139 (2)
N4—H4*B*···O3	0.87 (2)	2.06 (2)	2.909 (3)	166 (2)
N5—H5*A*···O5^ii^	0.89 (2)	1.98 (2)	2.871 (3)	176 (3)
N5—H5*B*···O2	0.88 (2)	1.99 (2)	2.863 (3)	169 (3)
N9—H9*A*···N7	0.86 (2)	1.98 (2)	2.683 (3)	138 (2)
N9—H9*B*···O5^v^	0.85 (2)	2.06 (2)	2.906 (3)	174 (2)
N10—H10*A*···O4^v^	0.88 (2)	2.00 (2)	2.863 (3)	167 (3)
N10—H10*B*···O3^iv^	0.89 (2)	1.99 (2)	2.872 (3)	179 (3)
C2—H2···O2	0.95	2.57	2.929 (3)	103
C7—H7*C*···O4^vi^	0.98	2.53	3.283 (3)	133
C20—H20···O4	0.95	2.57	2.928 (3)	102

Symmetry codes: (i) −*x*, *y*+1/2, −*z*+1/2; (ii) *x*−1, −*y*+1/2, *z*−1/2; (iii) *x*, −*y*+1/2, *z*+1/2; (iv) −*x*+1, *y*+1/2, −*z*+3/2; (v) −*x*+1, −*y*+1, −*z*+2; (vi) *x*, *y*, *z*−1.
